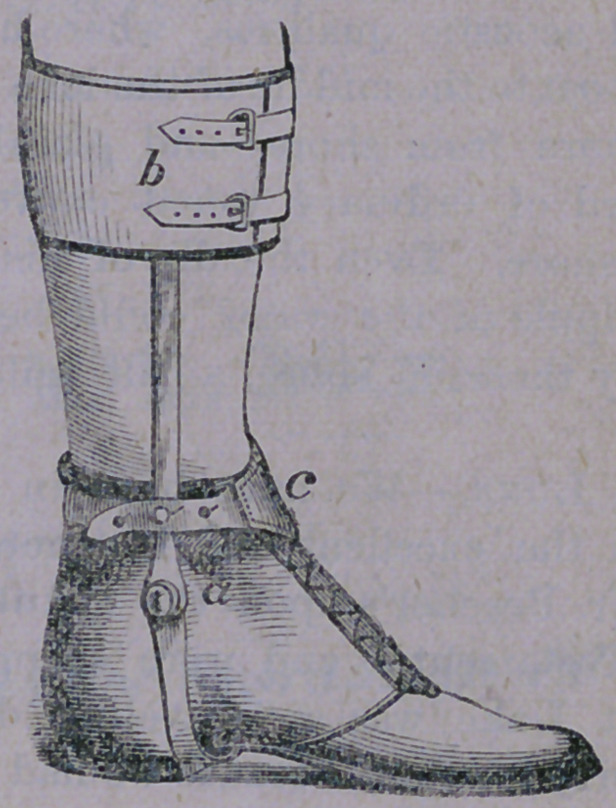# Deformed Feet

**Published:** 1875-01

**Authors:** 


### DEFORMED FEET.

In the October number of this journal, in 1873, we gave a description of one form of club-foot, called *talepes varus*, where the foot turns in such a manner as to cause the patient to step upon its *outer* side. We then described it fully, giving the means of cure. In the present article, we purpose treating of three other varieties of club-foot which the orthopædic surgeon is called upon to remedy.

The first is known as *talipes calcaneous*, and is that somewhat rare deformity where the child steps entirely upon the heel, the toes being elevated in the manner here illustrated:—

This deformity may arise from paralysis of the extensor muscles of the foot, when it will be found that the large tendon running to the heel, (*tendo achillis*) is soft and flaccid, while the muscles forming the "calf" of the limb are in like condition, giving the leg a shrivelled appearance. The muscles in front of the leg will be found to be intensely contracted, so that any attempt to bring the toes down to their natural position will be met with great resistance. The deformity may exist as a result of accident or disease, but is usually congenital.

In young children, the foot can be readily restored to its natural position by the application of the apparatus shown below:—

which is nothing more than a pair of steel rods, with suitable joints, riveted to a stout shoe; to the heel of which is fastened a lever, for the attachment of an elastic cord. This cord is again fastened to a band encircling the leg, by which also, the steel rods are supported. The elastic cord is lengthened or shortened, in order to afford the requisite tension, by means of the buckle, seen in the figure. By drawing this cord tight, the heel is necessarily drawn up, and the toes so thrown down. The continued strain of this cord upon the contracted muscles, tends to overcome them, and finally restores the foot to its normal position. When the case has been too long neglected, the tendons are required to be cut and the shoe then applied.

The second deformity is precisely the reverse of the first, and is named *talipes equinus*. As will be seen in the illustration, the heel is drawn up and the toes point in a direct continuation of the leg. In walking, the child steps upon the balls of the toes, the large one being drawn back, seemingly in order to give more surface for support. The large tendon (*tendo achillis*), is found to be very hard and tense, so that it is nearly impossible to draw the heel down to its natural position. If the deformity is allowed to exist for a considerable time, the foot becomes attenuated, and deformed, while that portion resting upon the floor becomes hard and caloused. This affection does not occur congenitally as frequently as the first difficulty. It is generally *acquired,* depending upon some affection of the spinal cord. It is often an accompaniment of spinal curvature. But whatever the *cause,* it is generally amenable to mechanical and surgical treatment. Division of the heel tendon is at once performed, when a shoe similar to the one here figured is applied:

An elastic band *e* is fastened to the sole of the shoe at *a,* while the other end is attached to a calf-band at *b.* The necessary degree of tension is regulated by shortening the band by means of the buckle *e.* The result from such an appliance will be so readily understood, and the illustration so easily comprehended, that further description is useless.

The third deformity of the foot which we have to describe is named *tapilas valgus.* This deformity consists in the turning of the sole of the foot *outward,* so that the patient walks upon the inner, or great-toe side of the foot.

This is, therefore, a very painful deformity, the skin soon becoming sore and ulcerated, so that locomotion is rendered very difficult, if not impossible. Sometimes this form of club-foot is caused by paralysis of the *tibial nerve,* and consequently of the internal (*adductor*) muscles of the foot. Again, it is produced by scrofulous inflammation of the articulating surfaces of the bones of the foot. It is remedied by means of an apparatus arranged as follows:

consisting of a stout leather shoe, with a stem riveted to its sole, and terminating in a joint at *a.* From this joint passes a stout steel bar fastened to a band encircling the calf of the leg, as at *b.* The foot is then kept in position by a triangular piece of leather fastened to the upright bar and encircling the ankle as illustrated at *c.*

We have thus taken pains to describe and plainly illustrate the various deformities of the feet, in order that parents can readily recognize them, and at once perceive how easily they can be corrected by means of suitable apparatus, in the possession of every skillful surgeon. It is to be hoped that parents will speedily avail themselves of the opportunities thus afforded them, for correcting any such difficulties that may chance to exist among their children, and not allow them to grow up miserably and helplessly deformed. The deformed feet here described, are readily curable in the young. The longer treatment is delayed, the less become the chances for recovery.

## Figures and Tables

**Figure f1:**
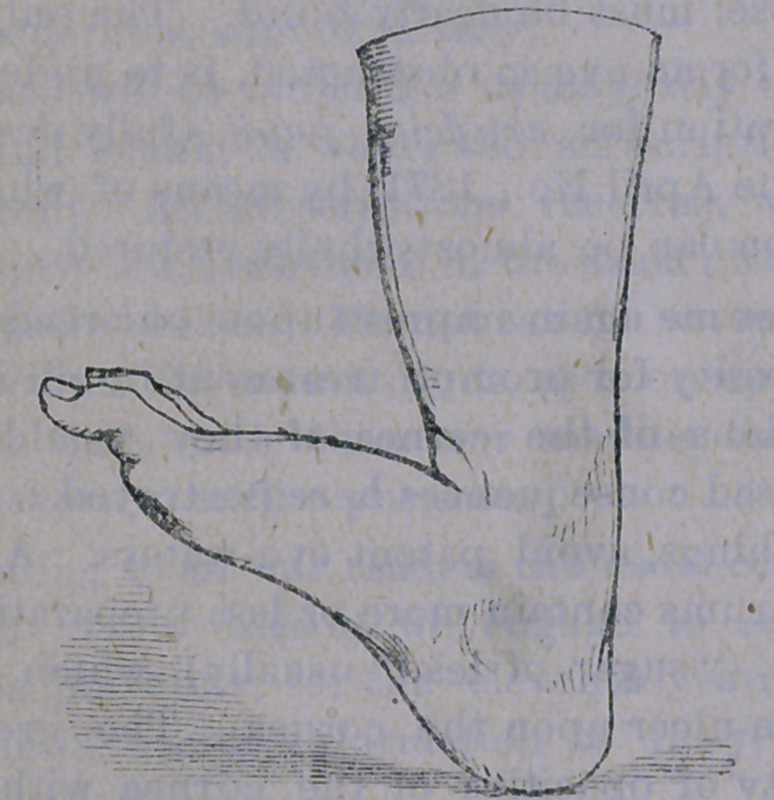


**Figure f2:**
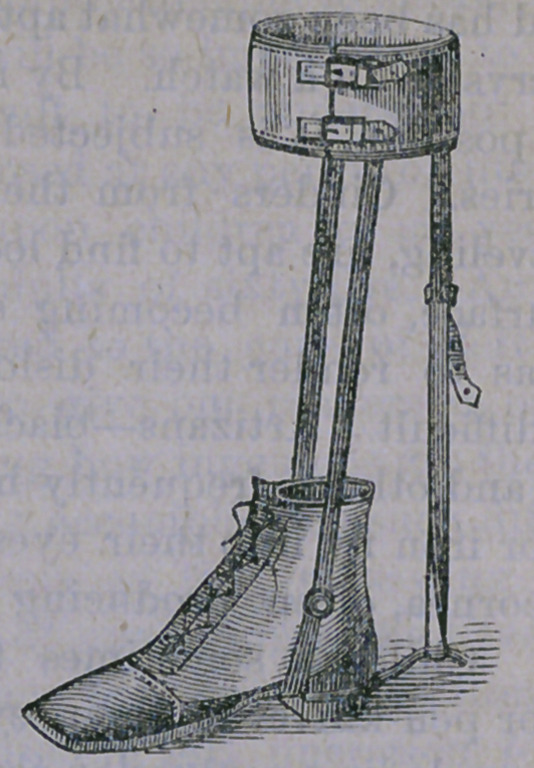


**Figure f3:**
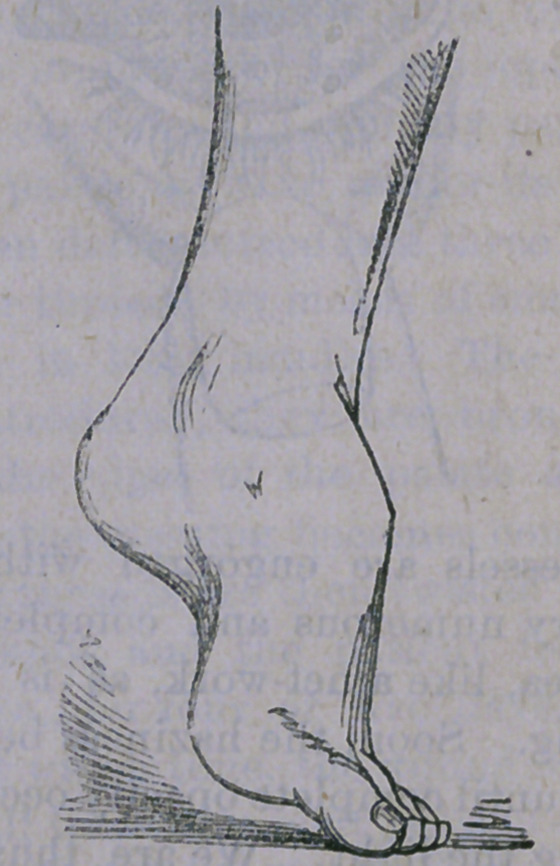


**Figure f4:**
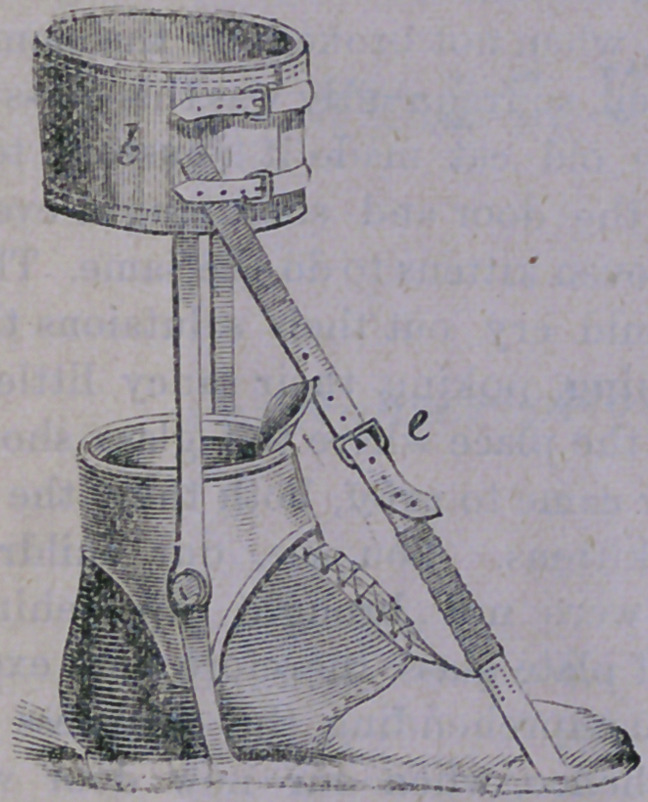


**Figure f5:**
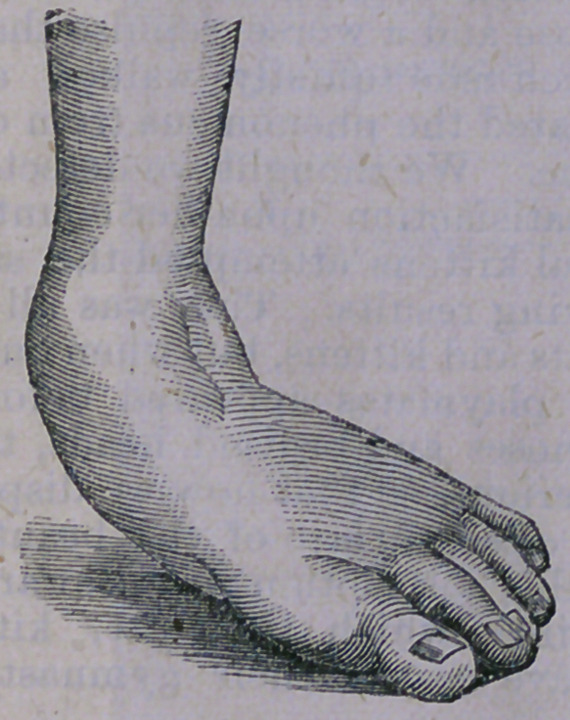


**Figure f6:**